# Application of CRISPR/Cas9 System for Plasmid Elimination and Bacterial Killing of *Bacillus cereus* Group Strains

**DOI:** 10.3389/fmicb.2021.536357

**Published:** 2021-06-10

**Authors:** Xiaojing Wang, Yufei Lyu, Siya Wang, Qingfang Zheng, Erling Feng, Li Zhu, Chao Pan, Shenghou Wang, Dongshu Wang, Xiankai Liu, Hengliang Wang

**Affiliations:** ^1^State Key Laboratory of Pathogens and Biosecurity, Beijing Institute of Biotechnology, Beijing, China; ^2^Experimental Teaching Center, Shenyang Normal University, Shenyang, China; ^3^College of Food Science and Technology, Shanghai Ocean University, Ministry of Agriculture Shanghai Engineering Research Center of Aquatic Product Processing & Preservation, Laboratory of Quality & Safety Risk Assessment for Aquatic Product on Storage and Preservation, Shanghai, China

**Keywords:** CRISPR/Cas9, sgRNA, *B. cereus* group, plasmid curing, virulence plasmid, sequence-specific antimicrobials

## Abstract

The CRISPR-Cas system has been widely applied in prokaryotic genome editing with its high efficiency and easy operation. We constructed some “scissors plasmids” via using the temperature-sensitive pJOE8999 shuttle plasmid, which carry the different 20nt (N20) guiding the Cas9 nuclease as a scissors to break the target DNA. We successfully used scissors plasmids to eliminate native plasmids from *Bacillus anthracis* and *Bacillus cereus*, and specifically killed *B. anthracis*. When curing pXO1 and pXO2 virulence plasmids from *B. anthracis* A16PI2 and A16Q1, respectively, we found that the plasmid elimination percentage was slightly higher when the sgRNA targeted the replication initiation region (96–100%), rather than the non-replication initiation region (88–92%). We also tried using a mixture of two scissors plasmids to simultaneously eliminate pXO1 and pXO2 plasmids from *B. anthracis*, and the single and double plasmid-cured rates were 29 and 14%, respectively. To our surprise, when we used the scissor plasmid containing two tandem sgRNAs to cure the target plasmids pXO1 and pXO2 from wild strain *B. anthracis* A16 simultaneously, only the second sgRNA could guide Cas9 to cleave the target plasmid with high efficiency, while the first sgRNA didn't work in all the experiments we designed. When we used the CRISPR/cas9 system to eliminate the pCE1 mega-virulence plasmid from *B. cereus* BC307 by simply changing the sgRNA, we also obtained a plasmid-cured isogenic strain at a very high elimination rate (69%). The sterilization efficiency of *B. anthracis* was about 93%, which is similar to the efficiency of plasmid curing, and there was no significant difference in the efficiency of among the scissors plasmids containing single sgRNA, targeting multi-sites, or single-site targeting and the two tandem sgRNA. This simple and effective curing method, which is applicable to *B. cereus* group strains, provides a new way to study these bacteria and their virulence profiles.

## Introduction

*Bacillus anthracis* is Gram-positive aerobic bacterium. It infects humans and animals through endospores dominant, causing anthrax. Anthrax is a rapidly-spreading malignant zoonotic disease with a short incubation period and a high mortality rate (Wui et al., 2013). *Bacillus anthracis* spores has been recognized as one of the three most dangerous biological weapons in the world, with potential to cause huge economic losses and bioterrorism (Weapon, 2002). *Bacillus anthracis* contains two virulence plasmids, pXO1 (181.6kb) and pXO2 (96.2kb), which encode the anthrax toxin and capsule, respectively (Ramisse et al., 1996). The pXO1 plasmid encodes anthrax toxin proteins such as the protective antigen (PA), the lethal factor, and the edema factor. The pXO2 plasmid encodes proteins involved in capsular biosynthesis and biodegradation (Levy et al., 2012). These two plasmids are critical to the pathogenicity of *B. anthracis*, and the loss of any one plasmid results in a great reduction in virulence (Agathe et al., 2003; Ariel et al., 2003). Therefore, eliminating the two virulence mega-plasmids and further examination of the pathogenic mechanism of *B. anthracis* will be important for the prevention and control of anthrax.

We sought to construct a plasmid-cured mutant strain for studying the role of plasmids in *B. anthracis*. In previous research of this area, the removal of bacterial plasmids involved chemical agents such as acridine orange, ethidium bromide, and high temperature culture or ultraviolet irradiation (Trevors, 1986), all of which have some potential problems. The first is the poor specificity; in other words, it is possible to drive out other plasmids, while the second may cause random mutations in the host chromosome during such treatments. Therefore, our laboratory used a small, high-copy plasmid to drive out plasmids based on the plasmid incompatibility principle (Wang et al., 2011; Liu et al., 2012). This method has a better specificity than the physical and chemical methods, but requires the exact information about the sequence of the origin of replication of the plasmid. This method is also time consuming.

*Bacillus cereus*, a Gram-positive opportunistic pathogen (Hauge, 1950), is widespread in soil, sewage, and all types of foods. *Bacillus cereus* produces a range of virulence factors, including enterotoxins and an emetic toxin that causes diarrhea and emetic types of food poisoning (Drobniewski, 1993; Arnesen et al., 2008). The data show that there have been several cases of severe and even fatal food poisoning caused by *B. cereus*. Cereulide, a heat-stable emetic toxin produced by the non-ribosomal peptide synthetase (NRPS) gene cluster on a large *B. cereus* plasmid (Ehling-Schulz et al., 2005), is thermostable (heat stable at 121°C for 20 min) and resistant to proteolytic degradation (Shinagawa et al., 1995; Agata et al., 1996). It is responsible for the emetic type of gastrointestinal disease caused by *B. cereus*; hence, curing this plasmid to construct mutants could help researchers to investigate food poisoning outbreaks from *B. cereus*.

*Bacillus cereus* is very closely related to *B. anthracis*; they are both the members of the *B. cereus* group, which is a term used to describe a genetically highly homogeneous subdivision of the *Bacillus* genus (Helgason, 2000). Plasmids are vitally important for the *B. cereus* group as virulence determinants. Therefore, establishing a rapid and efficient method to eliminate virulence plasmids in *B. cereus*-group strains would help to study the relationship between the virulence plasmid and chromosome, and develop a safer, more effective vaccines and drugs to prevent and treat the diseases caused by this group of bacteria.

In 2013, the CRISPR/Cas9 system was first used for genome editing of human and mouse embryonic stem cells. The Cas9 protein contains two nuclease domains that can cleave two strands of DNA (Bikard et al., 2012). Cas9 first combines with crRNA and tracrRNA to form a complex, and then binds to DNA through the protospacer adjacent motif (PAM) sequence to form an RNA–DNA complex structure and cleave the double DNA strands (Vercoe et al., 2013). As the PAM sequence is simple in structure (5′-NGG-3′), a large number of targets can be found in almost all genes, thus the CRISPR/Cas9 system is widely used (Bikard et al., 2014). It has been successfully applied to mice, pigs, zebrafish, arabidopsis, sorghum, nematodes, yeast, *Escherichia coli* and many other animals, plants and microorganisms, and has become a genome editing tool widely used in various fields of biology and medicine (Citorik et al., 2014). pJOE8999 is a CRISPR/Cas9 single plasmid system constructed by V. Müller, who used it for *B. subtilis* genome editing to construct mutants quickly and efficiently (Altenbuchner, 2016).

In this study, the CRISPR/Cas9 system was used to cure plasmids in *B. anthracis* and *B. cereus*, and specifically kill *B. anthracis*. We found that it provides a faster and more convenient experimental method for constructing a new vaccine strain compared with older methods and may also provide a new approach toward the control of *B. anthracis*.

## Materials and Methods

### Plasmids and Strains

The plasmids and strains used in this study are shown in Table 1.

**Table 1:** Plasmids and strains used in this study.

**Plasmids and Strains**	**Relevant genotype and characteristics**	**Source**
**Plasmids**		
pJOE8999	Contains CRISPR-Cas9 system plasmid for breaking genome; Kanamycin (30 μg/mL)	Altenbuchner, 2016
pJO1T	pJOE8999 plasmid containing sgRNA sequence target to the replication initiation region of pXO1 plasmid in *Bacillus anthracis (B. anthracis)*	This study
pJO2T	pJOE8999 plasmid containing sgRNA sequence target to the replication initiation region of pXO2 plasmid in *B. anthracis*	This study
pJO1NT	pJOE8999 plasmid containing sgRNA sequence target to the non-replication initiation region of pXO1 plasmid in *B. anthracis*	This study
pJO2NT	pJOE8999 plasmid containing sgRNA sequence target to the non-replication initiation region of pXO2 plasmid in *B. anthracis*	This study
pJN1F2T	pJOE8999 plasmid successively containing O1NT, ‘gRNA and O2T, the two sgRNA sharing with a promoter P_vanP^*^_.	This study
pJF2N1T	pJOE8999 plasmid successively containing O2T, ‘gRNA and O1NT, the two sgRNA sharing with a promoter P_vanP^*^_.	This study
pJN1F2W	pJOE8999 plasmid successively containing O1NT, ‘gRNA-ter, P_vanP^*^_ and O2T, the two sgRNA with the respective promoter P_vanP^*^_, the first sgRNA including terminator.	This study
pJF2N1W	pJOE8999 plasmid successively containing O2T,'gRNA-ter, P_vanP^*^_ and O1NT, the two sgRNA with the respective promoter P_vanP^*^_, the first sgRNA including terminator.	This study
pJ16ST	pJOE8999 plasmid containing sgRNA sequence target to 16S rRNA of *B. anthracis*	This study
pJART	pJOE8999 plasmid containing sgRNA sequence target to a specific DNA fragment of the chromosome of *B. anthracis*	This study
pJHNT	pJOE8999 plasmid containing sgRNA sequence target to a specific DNA fragment of the chromosome of *B. cereus*	This study
pJA16SRT	pJOE8999 plasmid containing two sgRNAs sequence targeting on 16S rRNA (16ST) and non-replication initiation region of a specific DNA fragment of the chromosome in *B. anthracis* (ART), the two sgRNA sharing with a promoter P_vanP^*^_.	This study
pJA16SRTW	pJOE8999 plasmid containing two sgRNAs sequence targeting on 16S rRNA and non- replication initiation region of a specific DNA fragment of the chromosome in *B. anthracis*, the two sgRNA with the respective promoter P_vanP^*^_, the first sgRNA including terminator.	This study
pJp1T	pJOE8999 plasmid containing sgRNA sequence target to pCE1 plasmid of *B. cereus* BC307	This study
**Strains**		
*B. anthracis* A16PI2	pXO2 plasmid-cured derivative of wild type A16 using Plasmid Incompatibility; pXO1^+^, pXO2^−^	Wang et al., 2011
*B. anthracis* pJO1T/A16PI2	A16PI2 contains recombinant plasmid pJO1T; pXO1^+^, pJO1T^+^	This study
*B. anthracis* A16PI2D1	pXO1 plasmid-cured derivative of A16PI2 using CRISPR/Case9 system; pXO1^−^	This study
*B. anthracis* A16Q1	pXO1 plasmid-cured derivative of wild type A16 using plasmid incompatibility; pXO1^−^, pXO2^+^	Liu et al., 2012
*B. anthracis* pJO2T/A16Q1	A16Q1 contains recombinant plasmid pJO2T; pXO2^+^ pJO2T^+^	This study
*B. anthracis* A16Q1D2	pXO2 plasmid-cured derivative of A16Q1 using CRISPR/Cas9 system; pXO2^−^	This study
*B. anthracis* A16	Wild type A16 isolated from the carcass of a mule that died from anthrax in Hebei Province, China, in 1953; pXO1^+^, pXO2^+^	This lab
*B. anthracis* pJO1TpJO2T/A16	A16 contains recombinant plasmid pJO1T and pJO2T; pXO1^+^pXO2^+^ pJO1T^+^ pJO2T^+^	This study
pJN1F2T/A16	A16 contains recombinant plasmid pJN1F2T: pXO1^+^pXO2^+^ pJN1F2T^+^	This study
pJF2N1T/A16	A16 contains recombinant plasmid pJF2N1T: pXO1^+^pXO2^+^ pJF2N1T^+^	This study
pJN1F2W/A16	A16 contains recombinant plasmid pJN1F2TW: pXO1^+^pXO2^+^ pJN1F2TW^+^	This study
pJF2N1W/A16	A16 contains recombinant plasmid pJF2N1TW: pXO1^+^pXO2^+^ pJF2N1W^+^	This study
*B. anthracis* A16MD1	pXO1 plasmid-cured derivative of wild type A16 using CRISPR/Cas9 system; pXO1^−^ pXO2^+^	This study
*B. anthracis* A16MD2	pXO2 plasmid-cured derivative of wild type A16 using CRISPR/Cas9 system; pXO1^+^ pXO2^−^	This study
*B. anthracis* A16MDD	pXO1 and pXO2 plasmid-cured derivative of wild type A16 using CRISPR/Cas9 system; pXO1^−^ pXO2^−^	This study
pJ16ST/A16PI2	A16PI2 contains recombinant plasmid pJ16ST	This study
pJART/A16PI2	A16PI2 contains recombinant plasmid pJART	This study
pJA16sRT/A16PI2	A16PI2 contains recombinant plasmid pJ16sRT	This study
pJA16sRTW/A16PI2	A16PI2 contains recombinant plasmid pJ16sRTW	This study
*B. cereus* HN001	*B. cereus* isolated from food poisoning	This lab
pJART/HN001	HN001 contains recombinant plasmid pJART	This study
*B. cereus* BC307	*B. cereus* isolated from the vomit of patients	This lab
pJp1T/BC307	BC307 contains recombinant plasmid pJp1T	This study
*B. cereus* BC307Dp1	pCE1 plasmid-cured derivative of BC307 using CRISPR/Cas9 system; pCE1^−^	This study

*The semisynthetic promoter. The sgRNA transcribed from the semisynthetic promoter P_vanP_*

* *interrupted by the lacZ α fragment (lacPOZ')*.

### Curing Plasmids From *B. anthracis*

#### Construction of “Scissors Plasmids” to Cure pXO1 and pXO2 Plasmids

We designed N20 sequences against specific sequences on pXO1 and pXO2 plasmids (GenBank accession Nos. NC_007323 and AF065404, respectively). The N20-specific target sequence (Table 2) in the single guide RNA (sgRNA) was designed via using sgRNAcas9_V3.0_GUI software (Xie et al., 2014). To evaluate whether this method is feasible when we did not know the precise origin of DNA replication, we designed the N20 sequences that target both the possible replication initiation region and the non-replication initiation region. The N20 sequences on pXO1 and pXO2 origins of DNA replication were named O1T and O2T, while the N20 sequences on the pXO1 and pXO2 non-origins of DNA replication were named O1NT and O2NT.

**Table 2:** Oligonucleotide sequences and primers used in this study.

**Name**	**Sequence**	**Description**
O1T and PAM:	ATAACTTGTAATAGCCCTTT**AGG**	N20 sequence and PAM on pXO1 origin of DNA replication
O2T and PAM:	ACACAAAGTGATAGCCTAGA**TGG**	N20 sequence and PAM on pXO2 origin of DNA replication
FO1T	TACG ATAACTTGTAATAGCCCTTT	O1T sequence 5′ end plus TACG connector
RO1T	AAAC AAAGGGCTATTACAAGTTAT	O1T reverse complementary sequence 5′ end plus AAAC linker
FO2T	TACG ACACAAAGTGATAGCCTAGA	O2T sequence 5′ end plus TACG connector
RO2T	AAAC TCTAGGCTATCACTTTGTGT	O2T reverse complementary sequence 5′ end plus AAAC linker
O1NTand PAM	TATTCGATGAAGTCATACAC**TGG**	N20 sequence and PAM on pXO1 non-origin of DNA replication
O2NTand PAM	CTACTTATAAGAACAAACCG**AGG**	N20 sequence and PAM on pXO2 non-origin of DNA replication
FO1NT	TACG TATTCGATGAAGTCATACAC	O1NT sequence 5′ end plus TACG connector
RO1NT	AAAC GTGTATGACTTCATCGAATA	O1NT reverse complementary sequence 5′ end plus AAAC linker
FO2NT	TACG CTACTTATAAGAACAAACCG	O2NT sequence 5′ end plus TACG connector
RO2NT	AAAC CGGTTTGTTCTTATAAGTAG	O2NT reverse complementary sequence 5′ end plus AAAC linker
16ST and PAM	CGTGAGTGATGAAGGCTTTC**GGG**	N20 sequence targeting the 16SrNA region of *B. anthracis*
ART and PAM	ACACGGATGATAATAATTTG**TGG**	*B. anthracis* specific N20 sequence
Spacer-F	AACCATCACTGTACCTCCCA	Two BsaI outer primers on pJOE8999, verifying whether N20 is successfully linked
Spacer-R	GAGCGTTCTGAACAAATCCA	
pJOE8999-F	TAGTGTAGCCGTAGTTAGG	Specific sequence primers on pJOE8999 to verify the presence of pJOE8999
pJOE8999-R	AAAGGGAATGAGAATAGTG	
cya-F	AGGATTGATGTGCTGAAAGGAG	cya gene primer pair on pXO1
cya-R	TTCGTCTTTGTCGCCACTATC	
pXO1-7F	CGTACTGCTGGAATTGATGG	A specific gene primer pair on pXO1
pXO1-7R	GTCTTGGCTAACACCTGTATG	
pXO1-13F	AGAAATTGAGTTTGAATATGGTGAG	A specific gene primer pair on pXO1
pXO1-13R	AGGTTGGCTTACTGGAGATAC	
pXO1-16F	AGCACATGACATACGAAGAAC	A specific gene primer pair on pXO1
pXO1-16R	GAACATAAGAAGTCTGAATGGATAG	
pXO1-23F	AACTAAGACACAACGAATACTACG	A specific gene primer pair on pXO1
pXO1-23R	CATTATGTGGTCAAGATTATGGTTC	
pXO1-32F	TGAACATGAACTAGAGGAATTGG	A specific gene primer pair on pXO1
pXO1-32R	ATCTTCTGGAGTCGGATTAGC	
pXO1-42F	ATCTGTGCTGCTCGTATCG	A specific gene primer pair on pXO1
pXO1-42R	GGAATCCTGGAATGAATGATGG	
pXO1-51F	TTGCCTGAGGTTCCTGTTG	A specific gene primer pair on pXO1
pXO1-51R	GCTTTCTCTCCCTTTGTGTAAG	
pXO1-55F	CGAATGAAGGTTATTGGAATAGC	A specific gene primer pair on pXO1
pXO1-55R	CTGGATCTGGATTAGGTGTTAC	
pXO1-59F	GGACTCGGAACAACAATAACG	A specific gene primer pair on pXO1
pXO1-59R	CCTCTCCATTTCGGCTGAC	
pXO1-67F	AATGGGAATCAAAGTTTACAATCTG	A specific gene primer pair on pXO1
pXO1-67R	ACTGAACACCACCTACCTTATC	
pXO1-70F	CATACCATTACAGGAGCATCATC	A specific gene primer pair on pXO1
pXO1-70R	ACCAGGAATCGCAAGAACC	
pXO1-90F	AAGGAAGTAGAGGCAGAAGC	A specific gene primer pair on pXO1
pXO1-90R	TTAATGTGTTGGCGTTCAGG	
pXO1-95F	GTCTATCAGAAGTAGGTCATAACG	A specific gene primer pair on pXO1
pXO1-95R	TTCAGTAAGAGCCTCCATAGTAG	
pXO1-98F	GACTGGTATTTCTACTGGGTTTG	A specific gene primer pair on pXO1
pXO1-98R	GTCCTGCTTCTTGATGATGATG	
pXO1-116F	CCTTCGTTCTGGTGATATGTG	A specific gene primer pair on pXO1
pXO1-116R	AATAATATGTGGTGCCTCTTCTG	
pXO1-133F	ATTGTGGAGGATAGATTCTTTGG	A specific gene primer pair on pXO1
pXO1-133R	TCTCGCTTGGCTAATTTCATC	
pXO1-142F	CGTGGACATCTGCTTGAAC	A specific gene primer pair on pXO1
pXO1-142R	GACGACCTTCCTCTTGATATTG	
capA-F	CGATGACGATGGGTGAC	capA gene primer pair on pXO2
capA-R	AGATTGAAGTACATGCGGATGG	
pXO2-007F	GCGATGGTGGAACAGGAATG	A specific gene primer pair on pXO2
pXO2-007R	TGCGTTGCTGCCGATATTG	
pXO2-016F	CGGTTTGGTATGAGTGAGGAAG	A specific gene primer pair on pXO2
pXO2-016R	ATTGGCTGTGGTGGTTGTTG	
pXO2-023F	TTGGGACAGGCGTTATAGAAAG	A specific gene primer pair on pXO2
pXO2-023R	GCAGCGAAGTCACTACATGG	
pXO2-027F	GTGGACTTCCTGTAACCGTAAG	A specific gene primer pair on pXO2
pXO2-027R	ATGTAATGGCTGCGTCACTTC	
pXO2-039F	GCTTCTCACTGGACACCTAATG	A specific gene primer pair on pXO2
pXO2-039R	CCACTCGTGCCAATGACTAC	
pXO2-060F	CGAAAGCAACAGGGATACAAAG	A specific gene primer pair on pXO2
pXO2-060R	AGATACTCTGCCCAACTTTCAC	
pXO2-084F	AGCGTTCAAATACAGTCACATC	A specific gene primer pair on pXO2
pXO2-084R	TTACCTTTGCGATTTCCTCATC	
pXO2-089F	AACTGACGGTGAATCCATGAAC	A specific gene primer pair on pXO2
pXO2-089R	ATTGCCTGACTAATCGCTAAGC	
pXO2-094F	CCTGGGCGTAAAGAAGATGG	A specific gene primer pair on pXO2
pXO2-094R	TCTCGTTGCGTGACATTATCG	
pXO2-097F	AAGCAACCCGTGGAGATTTC	A specific gene primer pair on pXO2
pXO2-097R	TGGATGTTCCGCACCTTTATAG	
pXO2-107F	TGGACGGAGAACAGGACTATG	A specific gene primer pair on pXO2
pXO2-107R	GGGCTTGCGGATACTCAGG	
pXO2-111F	ATACAAGCGAAGCATCAGTACC	A specific gene primer pair on pXO2
pXO2-111R	TCCATCGTTACAACCTCCATTC	
p1Tand PAM	AACTCCTAGTCAAGTACCAT**GGG**	N20 sequence and PAM on *B. cereus* BC307 pCE1 plasmid
Fp1T	TACG AACTCCTAGTCAAGTACCAT	p1T sequence 5′ end plus TACG connector
Rp1T	AAAC ATGGTACTTGACTAGGAGTT	p1T reverse complementary sequence 5′ end plus AAAC linker
P_vanP*_	GTGATTAGAGAATTGAGTAAAATGTACCTACG	The promoter from the pJOE8999 plasmid
‘gRNA	GCTAGAAATAGCAAGTTAAAATAAGGCTAGTCCGTTA TCAACTTGAAAAAGTGGCACCGAGTCGGTGCTTTTT	The gRNA from the pJOE8999 plasmid
Ter	ACTCCATCTGGATTTGTTCAGAACGCTC GGTTGCCGCCGGGCGTTTTTTATCTAAAGC TTAGGCCCAGTCGAAAGACTG	The terminator of opp from the pJOE8999 plasmid
cesA-F	TTCGGTGTTACTGTGTCTG	A specific gene (cesA gene) primer pair on *B. cereus* BC307 pCE1
cesA-F	ATCGCATTCTCTTCCATCC	
cesB-F	AACTTCAACCACAGGACAA	A specific gene (cesB gene) primer pair on *B. cereus* BC307 pCE1
cesB-R	ACATTACTATACCGCCAACA	
cesC-F	CATGTCGGCTATCTTCCAG	A specific gene (cesC gene) primer pair on *B. cereus* BC307 pCE1
cesC-R	GCAACCAGATTCTCCACTT	
cesD-F	GTGACAAGACCATTAGACC	A specific gene (cesD gene) primer pair on *B. cereus* BC307 pCE1
cesD-R	ACCTGAGACGATTAGTAGTA	
cesH-F	TCTGTTGTGGCAATAGGT	A specific gene (cesH gene) primer pair on *B. cereus* BC307 pCE1
cesH-R	GGAATGATAACTCCTTGACA	
cesP-F	AGGTGTGGATGTGGAGAA	A specific gene (cesP gene) primer pair on *B. cereus* BC307 pCE1
cesP-R	GATTGTCGGTCAGCCTAC	
cesT-F	CAGGCGGAAGTGCTAATG	A specific gene (cesT gene) primer pair on *B. cereus* BC307 pCE1
cesT-R	GTCCTCCTTCATAATGTATCAG	
p1-01F	AACCAAGCATACAGTCTCTT	A specific gene primer pair on *B. cereus* BC307 pCE1
p1-01R	CGTTGACCACTATCACCAT	
p1-02F	CGTTCTTATGTAGCCGATGT	A specific gene primer pair on *B. cereus* BC307 pCE1
p1-02R	GCTTCCTGTTATCACCACTA	
p1-03F	GGGTTTGGGTATCCGTAAT	A specific gene primer pair on *B. cereus* BC307 pCE1
p1-03R	ATGATTGGCGAAGAAGTGT	
p1-04F	CAGCACCTATCCAATTACCA	A specific gene primer pair on *B. cereus* BC307 pCE1
p1-04R	CATATTCAATCTCCATCCATCC	
p1-05F	CAGGAGACCAAGCACATC	A specific gene primer pair on *B. cereus* BC307 pCE1
p1-05R	CAAGAATATACTCGCTCAGAC	
p1-06F	GGTGGAGGAACAGGAACT	A specific gene primer pair on *B. cereus* BC307 pCE1
p1-06R	ATCGTCAGCAACTTCTACTT	
p1-07F	GAGAAGGCGATTGAAGGA	A specific gene primer pair on *B. cereus* BC307 pCE1
p1-07R	CCAGAGTGTAATGTCTTGTT	
p1-08F	CGAATAGCAGAGCCTGATAT	A specific gene primer pair on *B. cereus* BC307 pCE1
p1-08R	GGTAATCCAGAAGTGAATGTAG	

*The underlined part is the PAM sequence*.

Scissors plasmids were constructed using the plus TACG connector at 5′end of O1T and O2T sequences, and an AAAC linker at 5′end of O1T and O2T reverse complementary sequences, followed by synthesizing FO1T, RO1T, FO2T, RO2T, FO1NT, RO1NT, FO2NT, and RO2NT N20 oligonucleotides primers (Table 2). The two paired primers were annealed to obtain a double-stranded N20 oligonucleotide. The double-stranded N20 oligonucleotide product was inserted into pJOE8999 between the two *Bsa*I restriction sites. The detail of methods was shown S1 in **Supporting Information**. The recombinant plasmids were named pJO1T (from FO1T and RO1T primers), pJO2T (from FO2T and RO2T primers), pJO1NT (from FO1NT and RO1NT primers), and pJO2NT (from FO2NT and RO2NT primers) (Table 1).

**Figure 1:**
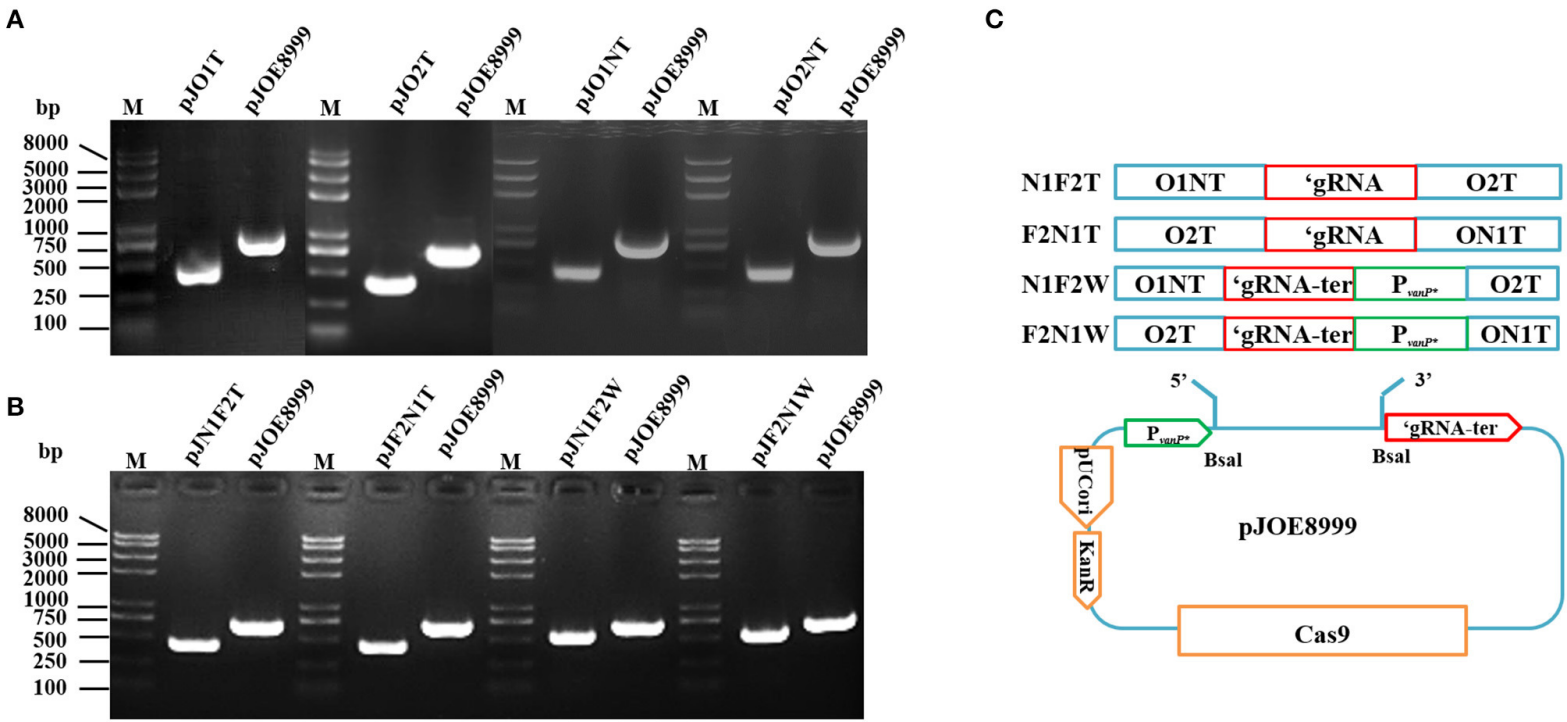
Colony PCR for pJO1T, pJO2T, pJO1NT, pJO2NT, pJN1F2T, pJF2N1T, pJN1F2W, and pJF2N1W constructs in *Escherichia coli* DH5α with primer pair spacer-F/R. M, *Trans*2K Plus II DNA marker. **(A)** The expected sizes of the fragments from pJO1T, pJO2T, pJO1NT, and pJO2NT are smaller than those from the pJOE8999 negative control. **(B)** The expected sizes of the fragments from pJO1T, pJO2T, pJO1NT, and pJO2NT are smaller than those from the pJOE8999 negative control. **(C)** The constructing process of the recombinant plasmid with two sgRNAs of O1NT and O2T inserted into pJOE8999 in tandem.

To investigate curing two plasmids by using tandem sgRNAs, we also designed a new recombinant scissors plasmid that inserted two tandem sgRNAs into the bone vector pJOE8999 to cure both pXO1 and pXO2 from wild type *B. anthracis* A16 simultaneously. The sequence N20_first_ – ‘gRNA-N20_second_ (or N20_first_-‘gRNA-ter-P_vanP*_-N20_second_) was inserted into the two *Bsa*I sites of pJOE8999 plasmid according to different order (Figure 1C). After ligation and transformation, PCR and sequencing were performed. The correctly constructed plasmids were named pJN1F2T, pJF2N1T, pJN1F2W, pJF2N1W, respectively.

#### Constructing Strains and Screening for Plasmid-Cured Colonies

We then transformed the demethylated scissors plasmids into *B. anthracis* by electroporation (500 Ω, 25 μF, 0.6 kV) (Shatalin and Neyfakh, 2005; Liu et al., 2012). For easy operation, we used attenuated strains of *B. anthracis* A16PI2 and A16Q1 to eliminate the pXO1 and pXO2, respectively (Table 1). The positive colonies selected by colony PCR with pJOE8999-F/R (Table 2) were designated pJO1T/A16PI2 (pXO1^+^ pJO1T^+^), pJO1NT/A16PI2 (pXO1^+^ pJO1NT^+^), pJO2T /A16Q1 (pXO2^+^ pJO2T^+^), and pJO2NT /A16Q1 (pXO2^+^ pJO2NT^+^).

Wild-type *B. anthracis*, harbors two large plasmids (pXO1 and pXO2) necessary for its complete virulence. To examine the two plasmids curing from *B. anthracis*, the demethylated plasmids pJO1T and pJO2T were equally mixed and simultaneously transformed into the virulent *B. anthracis* strain A16 (pXO1^+^, pXO2^+^; Table 1), and the positive colony recovered was designated pJO1TpJO2T /A16 (pXO1^+^ pXO2^+^ pJO1T^+^ pJO2T^+^).

These demethylated recombinant plasmids of pJN1F2T, pJF2N1T, pJN1F2W and pJF2N1W, containing two tandem sgRNAs targeting pXO1 and pXO2 respectively, were transformed into the virulent *B. anthracis* strain A16 (pXO1^+^, pXO2^+^; Table 1), the positive colonies recovered were designated pJN1F2T/A16, pJF2N1T/A16, pJN1F2W/A16, and pJF2N1W/A16 (Table 1).

The recombinant strains were grown in LB broth (containing 25 μg/ml kanamycin) at 30°C (220 rpm) for 3 h. The culture was added 0.4% D-mannose to induce Cas9 protein expression for 10 h. After induction of bacteria subculture (Altenbuchner, 2016), the plasmid-cured colonies were PCR-screened by diluting and plating the bacterial culture medium onto LB agar (containing 25 μg/ml kanamycin), and incubating it at 30°C overnight.

The pXO1-cured colonies were screened by colony PCR with *cya*-F/R primers, and authenticated using the other 17 specific primer pairs on pXO1 (Table 1). The pXO2-cured colonies were screened by colony PCR with *capA*-F/R primers, and authenticated using the other 12 specific primer pairs on pXO2 (Table 1).

We also used colony PCR with *cya*-F/R and *capA*-F/R primers to confirm the plasmid-cured strains from A16. We expected to obtain three types of strain from one experiment: a pXO1-cured strain, a pXO2-cured strain, and a dual plasmid-cured strain.

#### Eliminating the Scissors Plasmids

To eliminate the scissors plasmids, we passaged the plasmid-cured colonies twice at 37°C (220 rpm) in 5 mL of LB broth separately without antibiotics. Each passage culture was diluted and spread onto agar plates without antibiotics at 30°C for 12 h. We streaked single colonies onto two agar plates with or without kanamycin, and the kanamycin-sensitive colonies were the strains that had lost the scissors plasmids. We also used colony PCR with pJOE8999-F/R to verify elimination of the scissors plasmids (Table 2). The pXO1 plasmid-cured strain from A16PI2 was designated A16PI2D1 (pXO1^−^, pXO2^−^), the pXO2 plasmid-cured strain from A16Q1 was designated A16Q1D2 (pXO1^−^, pXO2^−^), the pXO1 plasmid-cured strain from A16 was designated A16MD1 (pXO1^−^, pXO2^+^), the pXO2 plasmid-cured strain from A16 was designated A16MD2 (pXO1^+^, pXO2^−^), and the pXO1 and pXO2 plasmid-cured strain from A16 were designated A16MDD (pXO1^−^, pXO2^−^).

#### Western Blot Analysis of the Anthrax Toxin PA

We used western blots to verify the curing of pXO1 from *B. anthracis* A16PI2 and A16. A16PI2 (pXO1^+^) and its derivative strain A16PI2D1 (pXO1^−^, pXO2^−^) comprised one group, and A16D1, A16D2, and A16DD strains comprised another group. The strains were inoculated into 50 ml of BHI broth (containing 5% horse serum and 0.8% NaHCO3) at 37°C in a 5% CO2 incubator for 13 h. The supernatant of culture was filtered through a 0.22 μm filter, and the solution was precipitated by a 3-times volume of precooled acetone for 3 h at 4°C. The acetone was evaporated, and the protein pellet dissolved in an appropriate amount of urea solution (50–200 μl) containing 8M urea, and 1% DTT. After quantification via the Bradford method, the protein samples were analyzed by SDS-PAGE and Western blots. The membrane was successively incubated with the mouse anti-PA monoclonal antibody diluted in TBST (1:25,000) for 1 h and the horseradish peroxidase (HRP)-labeled goat anti-mouse IgG (1:5,000) for 1 h. The membrane was uniformly covered with ECL luminescent liquid and photographed with a low-temperature gel imager (Kodak RP X-OMAT, USA).

#### Indian Ink Staining

The genes encoding capsule proteins are on the pXO2 plasmid and the pXO2-cured strain does not form the capsule. We used Indian ink staining to detect the capsule formation. A16Q1 (pXO2^+^) and A16Q1D2 (pXO2^−^) were one group, and A16, A16MD2 and A16MDD were the other group. The strains were inoculated onto an LB agar plate (containing 0.8% NaHCO_3_, 5% horse serum), under 5% CO_2_ at 37°C overnight. A loop of bacteria was inoculated into normal saline, a drop of Indian ink was added, and the strains were checked by phase-contrast microscopy (Eclipse TE300, Nikon, Tokyo, Japan) after squashing.

### Plasmid Curing in *B. cereus*

*Bacillus anthracis* and *B. cereus* belong to the *B. cereus* group. This group of bacteria is mostly plasmid containing, with the bacterial virulence gene mostly located on the plasmid. We used the plasmid curing method for the same purpose as in the other members of the *B. cereus* group. This involved a clinical strain of *B. cereus* BC307(CMCC(B) 63317), which was isolated from the vomit of a patient with food poisoning. After sequencing the whole genome by single molecule real-time sequencing using platform PacBio RS II, we found that the strain contains a 270 kb plasmid (pCE1), and that the NRPS gene cluster of the emetic cereulide toxin is located on this plasmid.

We designed the N20 sequence to target the pCE1 plasmid with the same method as above described to cure the pCE1 plasmid using the CRISPR/Cas9 system. We named this recombinant plasmid p1T. The construction method and the plasmid curing procedure were consistent with that used for *B. anthracis*. Scissors plasmid p1T was introduced into BC307 by electroporation to cure the pCE1 plasmid. After subculturing at 37°C to lose the p1T scissors plasmid, we preliminarily screened for pCE1 cured strains by colony PCR with *ces*-F/R, and further confirmed the curing of the pCE1 plasmid with 12 primer pairs (Table 2).

### Chromosome Targeting to Specifically Kill *B. anthracis* Using the CRISPR-Cas9 System

#### Construction of Plasmids and Strains

We designed the N20 sequence to target the bacterial chromosome for the purpose of sterilization, which might be a new way to prevent anthrax. We designed two types of N20 sequences for specific targeting of single-site and multi-sites (16S rRNA region) on the *B. anthracis* chromosome. The constructed plasmids were confirmed by PCR and sequencing and named pJART and pJ16ST (Table 1). The two plasmids were transformed into *E. coli* SCS110 for demethylation. We then transformed the extracted plasmids into *B. anthracis* A16PI2 and *B. cereus* HN001 by electroporation, and designated the constructed strains pJ16ST/A16PI2, pJART/A16PI2, and pJART/HN001 (Table 1).

According to the logical idea of Figure 1C, the N20 sequences of 16S rRNA and ART were together inserted into the pJOE8999 in tandem, and the constructed plasmids were transformed into *B. anthracis* A16PI2, named the strains pJA16sRT/A16PI2 and pJA16sRTW/A16PI2.

#### Sterilization Efficiency Determination Using the Colony Counting Method

To evaluate the sterilization efficiency of the two plasmids (pJART and pJ16ST), the constructed strains pJ16ST/A16PI2 and pJART/A16PI2 were cultured at 28°C for 3 h, 0.4% D-mannose was added (Altenbuchner, 2016), and the culturing was continued for 12 and 24 h, respectively. Strains cultured without D-mannose were the control group. Each bacterial culture was diluted (10^−1^-10^−6^) and 10 μL of each one was spread onto LB agar plates to compare the number of viable bacteria.

The killing efficiency of *B. anthracis* using the recombinant plasmid with two sgRNAs of 16ST and ART inserting into pJOE8999 was assessed by the method mentioned above.

The pJART/A16PI2 and pJART/HN001 strains were cultured in LB broth (containing 25 μg/mL kanamycin) for one generation, and sub-cultured for 3 h. The second-generation cultures of the two strains were mixed and inoculated into fresh LB medium at the same concentration. D-mannose (0.4%) was added to the induced group after 3 h. The mixed cultures were diluted (10^−6^) and spread onto LB agar plates (containing 0.5% yolk lotion, which is similar to Mannitol-Egg-Yolk- Polymyxin Agar Base) for 24 h. The specific killing efficiency of *B. anthracis* was determined as the colony forming units (CFU) for *B. anthracis* and *B. cereus*, as separately based on milky rings around the colonies on the agar plates.

#### Growth Curve Assays

The pJ16ST/A16PI2 and pJART/A16PI2 strains were inoculated into 5 mL LB broth (containing 25 μg/ml kanamycin) for 24 h at 37°C (220 rpm). Next, 1% of pJ16ST/A16PI2 and pJART/A16PI2 inoculum were subcultured into 35 mL of BHI broth and the optical density (OD value) was measured by the high-throughput real-time Microbial Analysis system (Gering Scientific Instruments Co. Ltd, Tianjing, China) at 37°C and 550 rpm. After 3 h, the broth of experimental group was added 0.4% D-mannose followed by monitoring for 24 h. Three independent biological repeats were performed.

#### Specifically Killing *B. anthracis* in *B. anthracis* and *B. cereus* Mixed Culture

The pJART/A16PI2 and pJART/HN001 strains were cultured in LB broth (containing 25 μg/mL kanamycin) for one generation, and sub-cultured for 3 h. The second-generation cultures of the two strains were mixed and inoculated into fresh LB medium at the same concentration. D-mannose (0.4%) was added to the induced group after 3 h. The mixed cultures were diluted (10^−6^) and spread onto LB agar plates (containing 0.5% yolk lotion, which is similar to Mannitol-Egg-Yolk- Polymyxin Agar Base) for 24 h. The specific killing efficiency of *B. anthracis* was determined as the colony forming units (CFU) for *B. anthracis* and *B. cereus*, as separately based on milky rings around the colonies on the agar plates.

## Results

### Plasmid Curing in *B. anthracis*

#### Identifying the Scissors Plasmids

Colony PCR was performed with spacer-F/R, and the scissors plasmid was successfully constructed. After O1T, O2T, O1NT, O2NT, N1F2T, F2N1T, N1F2W, and F2N1W were inserted into the temperature-sensitive pJOE8999 (7.8 Kb) shuttle plasmid, the amplified fragment was smaller than that of the backbone plasmid, and the DNA gel electrophoresis and DNA sequencing results showed that the “scissors plasmid” was successfully constructed (Figure 1). The scissors plasmids were named pJO1T, pJO2T, pJO1NT, and pJO2NT, pJN1F2T, pJF2N1T, pJN1F2W, and pJF2N1W, respectively.

#### Identifying the Constructed Strains

**Figure 2:**
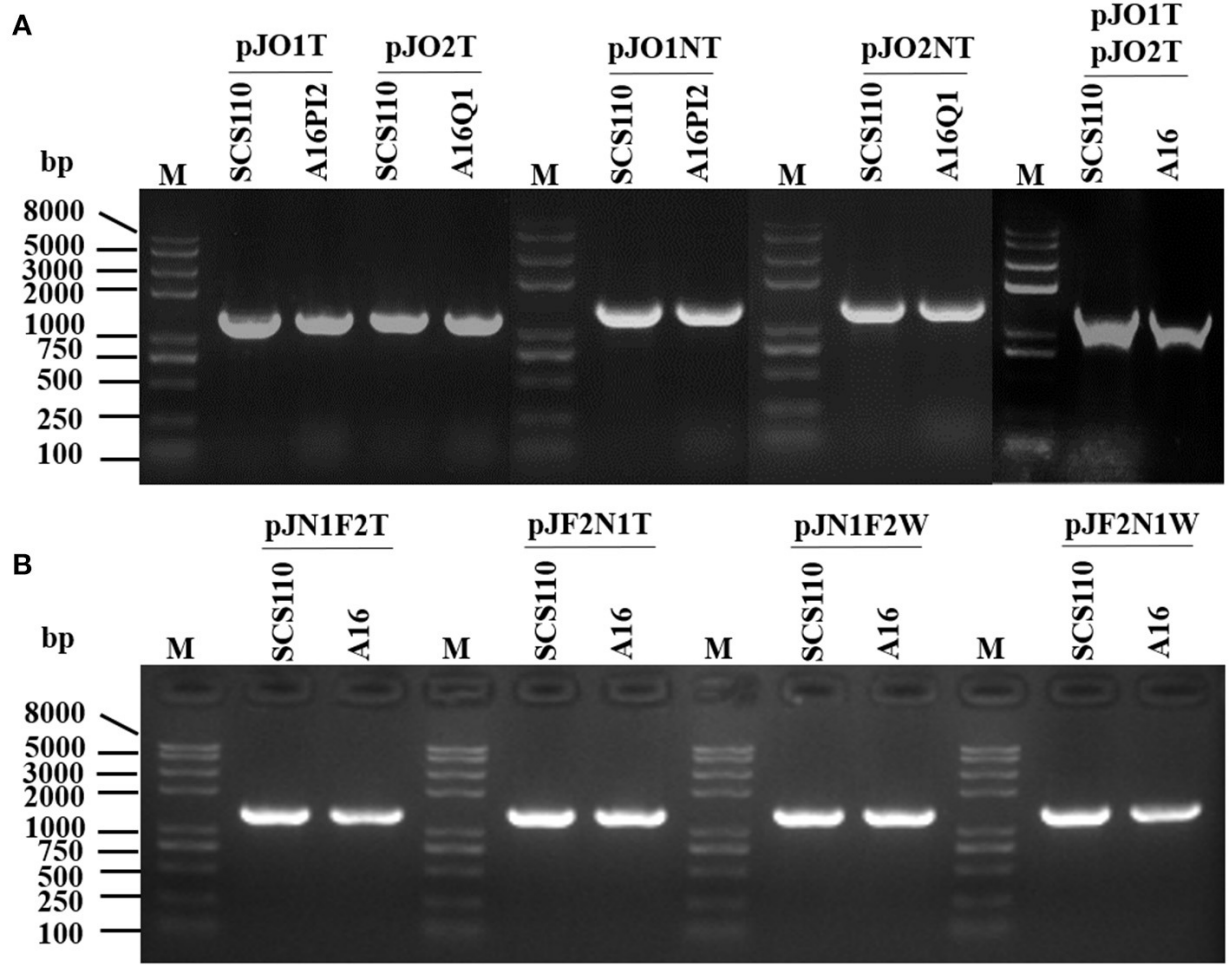
Colony PCR for screening scissors plasmid transformants in *Bacillus anthracis* with primer pair pJOE8999-F/R. M, *Trans*2K Plus II DNA marker. **(A,B)** *Escherichia coli* SCS110 containing a recombinant plasmid was the active control. The expected sizes of the fragments from the constructed strains are consistent with the size of the amplified fragments from the active control strain.

We identified the transformed *E. coli* DH5α, SCS110, and *B. anthracis* (A16PI2, A16Q1, A16) strains by colony PCR with pJOE8999-F/R primers. The result indicated that the scissors plasmid was successfully transformed into each strain (Figure 2). The constructed strains were named pJO1T/A16PI2 (pXO1^+^ pJO1T^+^), pJO2T /A16Q1 (pXO2^+^ pJO2T^+^), pJO1NT/A16PI2 (pXO1^+^ pJO1NT^+^), pJO2NT /A16Q1 (pXO2^+^ pJO2NT^+^), pJO1TpJO2T /A16 (pXO1^+^ pXO2^+^ pJO1T^+^ pJO2T^+^), pJN1F2T/A16(pXO1^+^ pXO2^+^ pJN1F2T^+^), pJF2N1T/A16(pXO1^+^ pXO2^+^ pJF2N1T^+^), pJN1F2W/A16(pXO1^+^ pXO2^+^ pJN1F2W^+^), and pJF2N1W/A16(pXO1^+^ pXO2^+^ pJF2N1W^+^).

#### Colony PCR Screening to Identify Plasmid Curing

**Figure 3:**
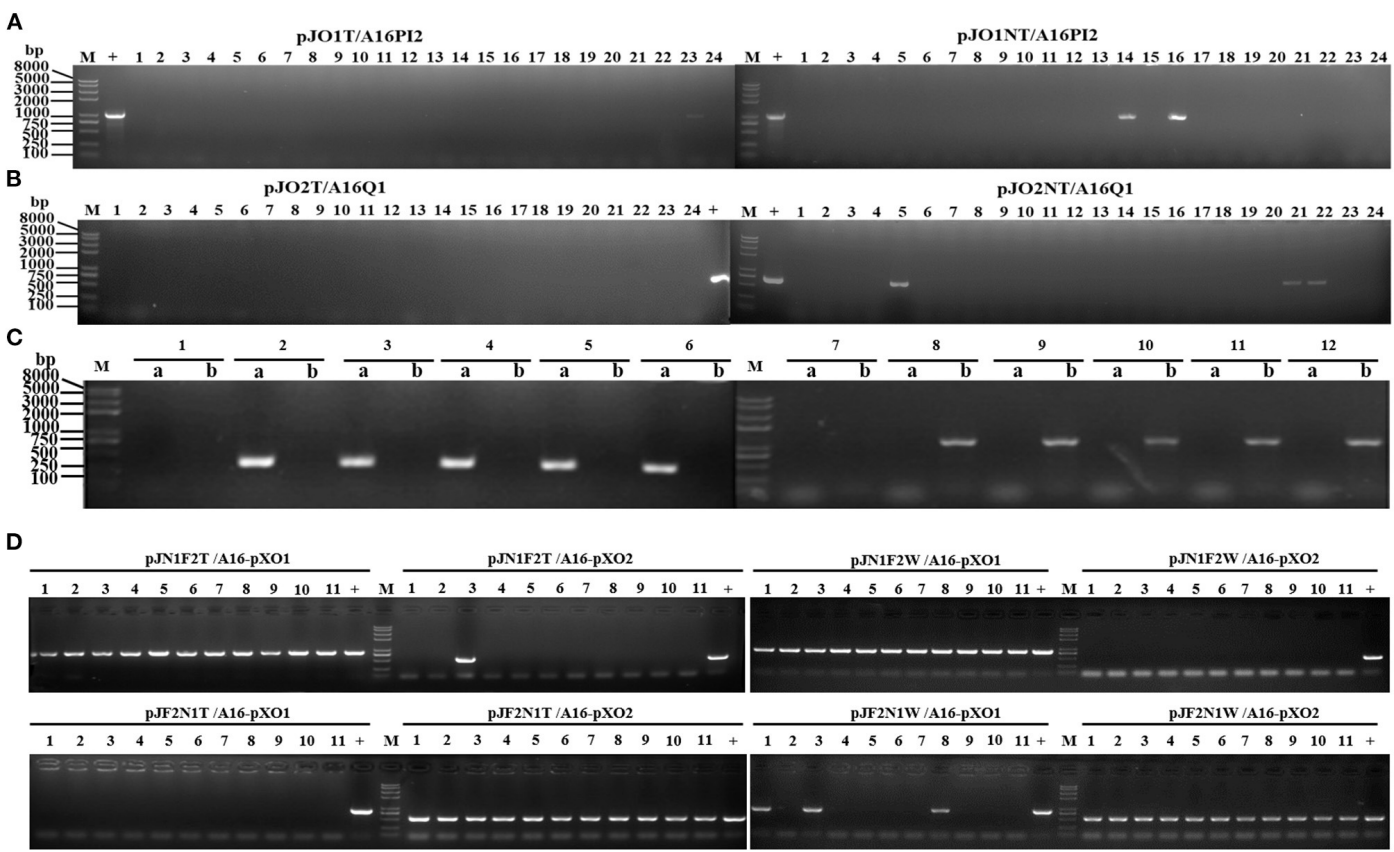
Preliminary PCR screening of colonies for pXO1 and pXO2 plasmid-cured strains with *cya*-F/R and *capA*-F/R primers, respectively. **(A)** Colony PCR with *cya*-F/R after curing the pXO1 plasmid from pJO1T/A16PI2 and pJO1NT/A16PI2. The pXO1 plasmid-cured strains do not have a specific amplification fragment, and the efficiency of pJO1T was about 96% (expect lane 23). The efficiency of pJO1NT was about 92% (expect lanes 14, 16). **(B)** Colony PCR with capA-F/R after curing the pXO2 plasmid from pJO2T/A16Q1 and pJO2NT/A16Q1. The pXO2 plasmid-cured strains do not have a specific amplification fragment, and the efficiency of pJO2T was about 100%. The efficiency of pJO2NT was about 88% (except lanes 5, 21, 22). **(C)** Colony PCR with *capC*-F/R (lane a) and *pag*-F/R primers (lane b) to screen for plasmid-cured strains from A16. The simultaneously cured pXO1 and pXO2 plasmids in A16 do not have specific amplification fragments in lanes a and b (1 and 7 monoclonal colonies). The pXO1 plasmid-cured strains in A16 do not have a specific amplification fragment in lane b but have a specific amplification fragment in lane a (2–6 monoclonal colonies). The pXO2 plasmid-cured strains in A16 do not have a specific amplification fragment in lane a, but have a specific amplification fragment in lane b (8–12 monoclonal colonies). **(D)** The efficiency of curing plasmids from *B. anthracis* A16 by using the recombinant plasmid with two sgRNAs of O1NT and O2T inserted into pJOE8999 in tandem. Colony PCR was revealed for pXO1 and pXO2 plasmid-cured strains with *cya*-F/R and *capA*-F/R. After with 0.4% D-mannose, the random 11 clones of pJN1F2T/A16, pJN1F2W/A16, pJF2N1T/A16, and pJF2N1W/A16 were used to assaying the curing rate. The curing pXO2 efficiency of pJN1F2T/A16 and pJN1F2W/A16 were respectively ~91 and 100%, and they both do not cure the pXO1. The curing pXO1 efficiency of pJF2N1T/A16 and pJF2N1W/A16 were respectively ~100 and 72%, and they both do not cure the pXO2. M, *Trans*2K Plus II DNA marker; lane1-11, 11 samples; +, control.

We used colony PCR to preliminarily screen for pXO1-cured strains from A16PI2 with *cya*-F/R primers by the scissor plasmid pJO1T or pJO1NT. The results indicated the 96% (23/24) and 92% (22/24) clones had eliminated pXO1 (Figure 3A). We used colony PCR to preliminarily screen for pXO2-cured strains from A16Q1 with primers *capA*-F/R by the scissor plasmids pJO2T or pJO2NT, the results of which indicated that 100% (24/24) and 88% (21/24) clones had eliminated pXO2 (Figure 3B). The results using the mixture plasmids of pJO1T and pJO2T to eliminate pXO1 and pXO2 from wild type strain A16 indicated that 5 clones (lanes 2–6) had eliminated the pXO1 plasmid, another 5 clones (lanes 8–12) had eliminated the pXO2 plasmid, and 2 clones (lanes 1 and 7) had eliminated both pXO1 and pXO2 (Figure 3C).

In the experiments of using two tandem sgRNAs to simultaneously cure the pXO1 and pXO2 in *B. anthracis* A16, the colony PCR was performed to assessed the curing efficiency with *cya*-F/R primers for the pXO1 and *capA*-F/R primers the pXO2. The 91% (10/11) and 100% (11/11) clones had eliminated pXO2 but not eliminated pXO1 by the pJN1F2T and the pJN1F2W, respectively. And the 100% (11/11) and 73% (8/11) clones had eliminated pXO1 but not eliminated pXO2 by the pJF2N1T and the pJF2N1W, respectively (Figure 3D). Our experimental results show that the recombinant scissors plasmid containing two tandem sgRNAs cannot simultaneously excise two target plasmids. The second sgRNA sequence could cure the corresponding target plasmid with high efficiency, but the first sgRNA didn't work in all the experiments we designed.

#### Elimination of Exogenous Scissors Plasmids

**Figure 4:**
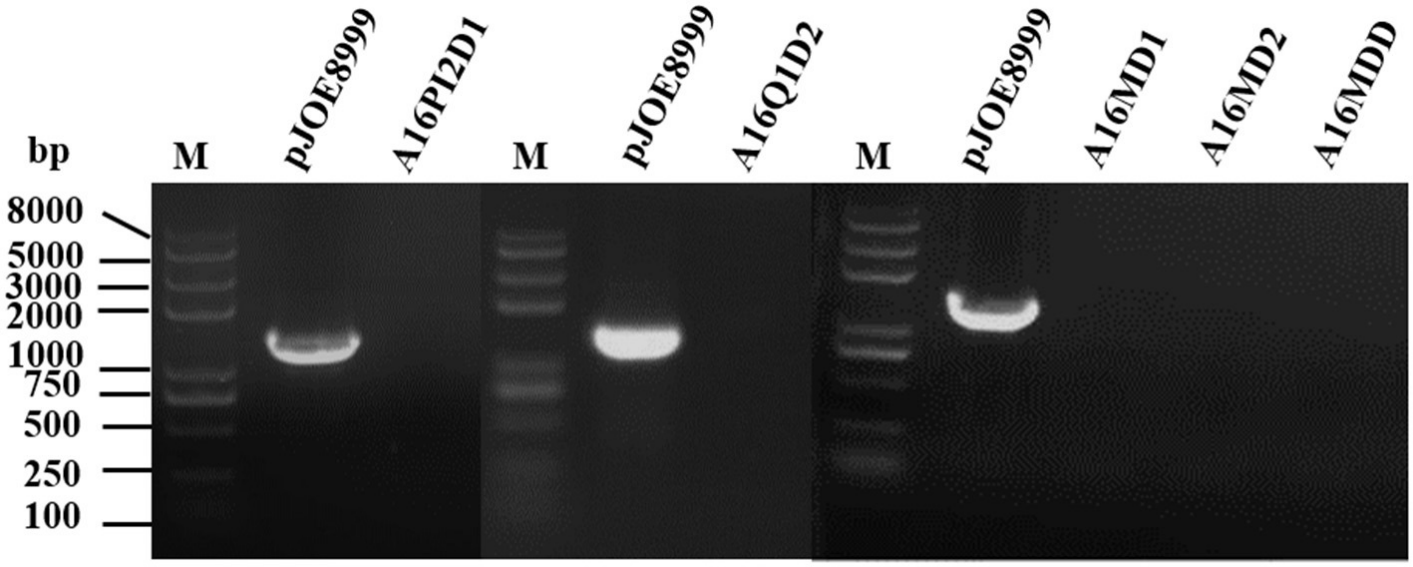
Agarose gel electrophoretogram of colony PCR to confirm the successful elimination of the exogenous scissors plasmid. M, *Trans*2K Plus II DNA marker. The exogenous scissors plasmid-eliminated strains (A16PI2D1, A16Q1D2, A16MD1, A16MD2, and A16MDD) lack specific amplification fragments, while the active pJOE8999 control plasmid generated specific amplification fragments.

We used colony PCR to identify strains where the exogenous scissors plasmid had been eliminated by using pJOE8999-F/R primers (Figure 4). The strains that had eliminated the scissors plasmid were designated A16PI2D1, A16Q1D2, A16MD1, A16MD2, and A16MDD.

#### Confirmation of Plasmid Curing

**Figure 5:**
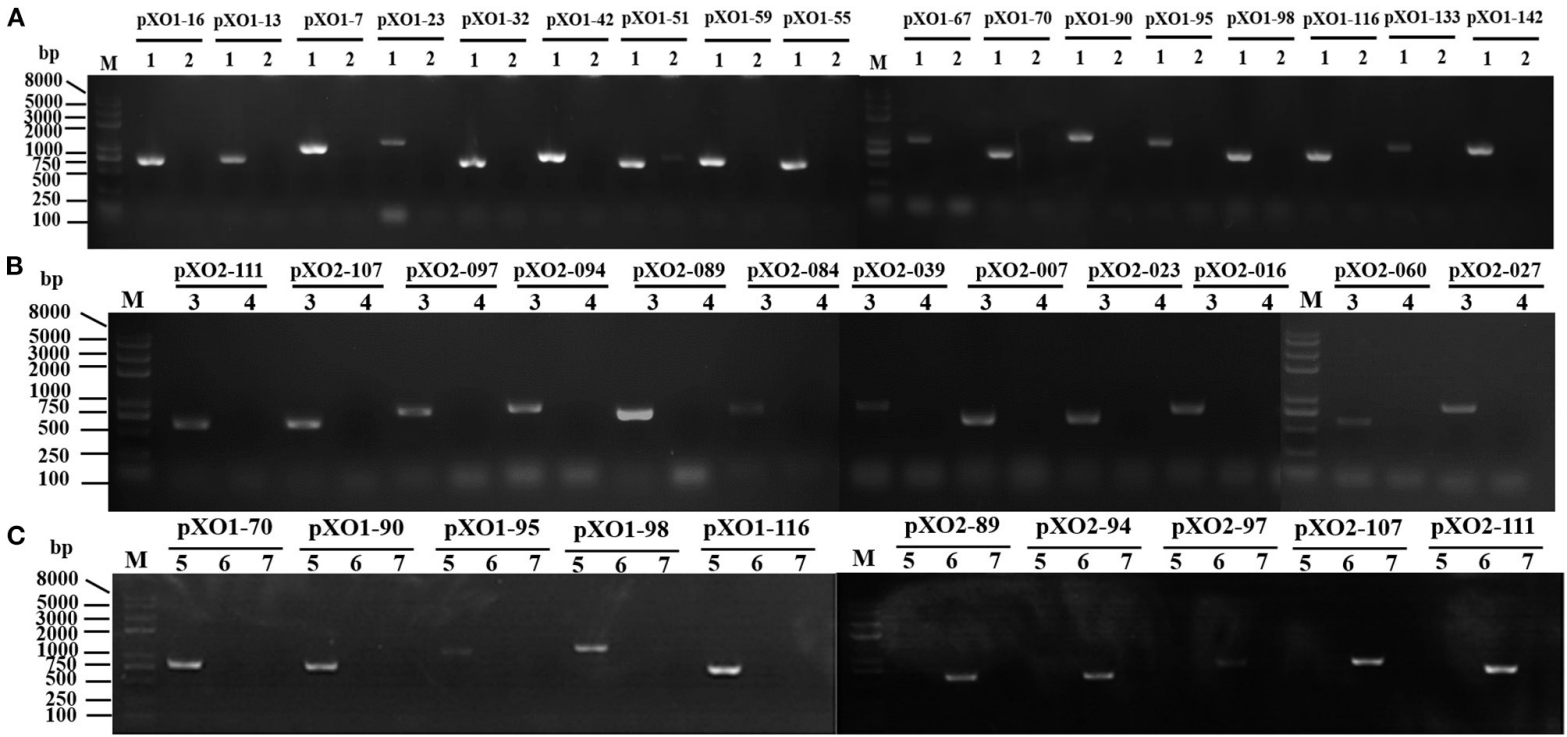
Agarose gel electrophoresis of colony PCR to identify plasmid curing via multiple primers. **(A)** The authenticity of the A16PI2D1 pXO1-cured strain was confirmed using 17 primer pairs. “pXO1-X” is used to represent the 17 gene primers on the pXO1 plasmid. A16PI2D1 (lane 2) lacks specific amplification fragments, while the A16PI2 control (lane 1) generated specific amplification fragments. **(B)** The authenticity of the pXO2-cured A16Q1D2 strain was confirmed using 12 primer pairs. “pXO2-X” is used to represent the 12 gene primers on the pXO2 plasmid. A16Q1D2 (lane 4) lacks specific amplification fragments, while the A16Q1 control (lane 3) generated specific amplification fragments. **(C)** Plasmids cured from A16 were authenticated using pXO1-X primers and pXO2-X primers. The pXO2-cured A16MD2 (pXO1^+^pXO2^−^) strain (lane 5) lacks specific amplification fragments from pXO2-X primers, but has specific amplification fragments from pXO1-X primers. The pXO1-cured A16MD1(pXO1^−^pXO2^+^) strain (lane 6) lacks specific amplification fragments from pXO1-X primers, but has specific amplification fragments from pXO2-X primers. The pXO1 and pXO2 simultaneously-cured A16MDD (pXO1^−^ pXO2^−^) strain (lane 7) lacks specific amplification fragments from pXO1-X primers and pXO2-X primers.

Colony PCR was used this time with multiple primers to identify the cured plasmid. The A16PI2D1 pXO1-cured strain was PCR-amplified with 17 pairs of primers (collectively called pXO1-X) and the results confirmed that A16PI2D1 lacks the pXO1 plasmid (Figure 5A). The pXO2-cured A16Q1D2 strain was PCR-amplified with 12 pairs of primers (collectively called pXO2-X) and the results indicated that A16Q1D2 lacks the pXO2 plasmid (Figure 5B). A16MD1, A16MD2, and A16MDD were also PCR-amplified with 5 pairs of primers (pXO1-X) and 5 other pairs of primers (pXO2-X) and the results indicated that A16MD1 lacks the pXO1 plasmid, A16MD2 lacks the pXO2 plasmid, and A16MDD lacks both pXO1 and pXO2 plasmids (Figure 5C). Thus, we successfully cured the target plasmid from the corresponding strains.

#### Western Blot Analysis and Indian Ink Dyeing

**Figure 6:**
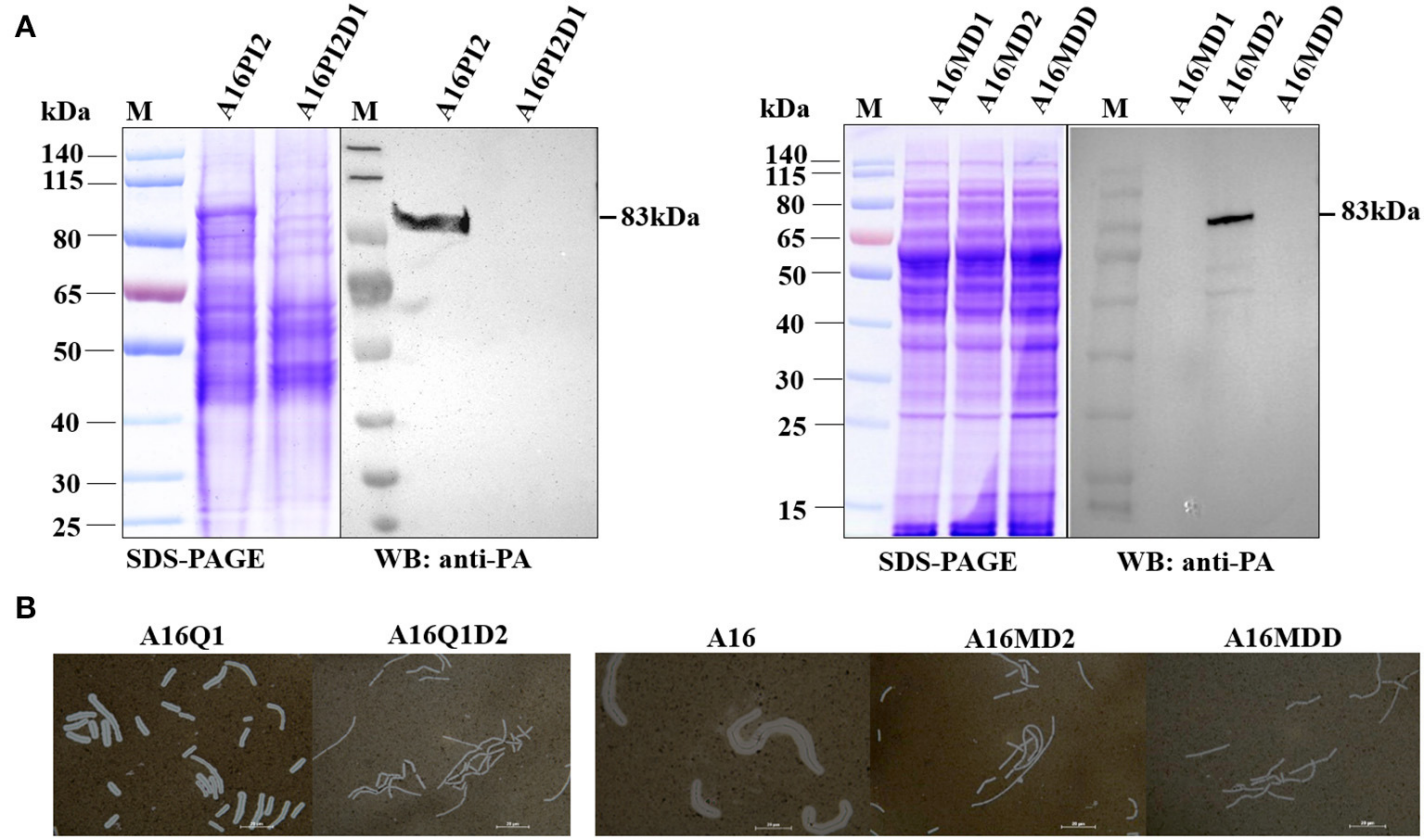
Difference between the active strain and the plasmid-cured strain. **(A)** Western blot detection of PA expression. M, Prestained protein ladder (PageRuler Prestained Protein Ladder, Product#26616; Thermo Fisher Scientific, Vilnius, Lithuania). SDS-PAGE assay and anti-PA western blotting with two sets of samples: A16PI2 (pXO1^+^) and A16PI2D1 (pXO1^−^), and A16MD1 (pXO1^−^), A16MD2 (pXO1^+^), and A16MDD (pXO1^−^). A16PI2D1 (pXO1^−^) lacks the anti-PA band whereas A16PI2 (pXO1^+^) has it. A16MD1 (pXO1^−^) and A16MDD (pXO1^−^) lack the anti-PA band whereas A16MD2 (pXO1^+^) has it. **(B)** Results of bacterial capsule Indian ink staining. One set of samples were A16Q1 and A16Q1D2, and the other were A16, A16MD2, and A16MDD. After dyeing with Indian ink, the background was gray and black. A16Q1D2 lacked any colorless transparent circles around the gray-colored bacteria, whereas A16Q1 had them. A16MD2 and A16MDD lacked any colorless transparent circles around the gray-colored bacteria, whereas A16 had them. pXO2-cured A16Q1D2, A16MD2 and A16MDD lacked capsular structures.

The pXO1 plasmid-containing strain was able to express the PA protein via the *pag* gene, whereas the pXO1 plasmid-cured one could not. Western blotting showed that A16PI2 (pXO1^+^) and A16MD2 (pXO1^+^ pXO2^−^) both expressed PA (83 kD) protein, whereas A16PI2D1 (pXO1^−^), A16MD1 (pXO1^−^ pXO2^+^), and A16MDD (pXO1^−^ pXO2^−^) did not (Figure 6A). Therefore, the pXO1 plasmid was cured in these strains.

The genes encoding the capsular protein are on the pXO2 plasmid. After the pXO2 plasmid was cured, the strain did not have a capsular structure. After Indian ink dyeing, the background was gray and black, and A16Q1 and A16MD1 contained the pXO2 plasmid and capsule structure, and a colorless transparent circle around the bacteria was visible against the gray background (Figure 6B). A16Q1D2, A16MD2, and A16MDD were cured of pXO2 plasmids, as revealed by the lack of colorless transparent circles, a well-known feature of the capsule structure (Figure 6B). These results indicate that plasmid pXO2 was cured in these strains.

### Curing the pCE1 Plasmid in *B. cereus*

**Figure 7:**
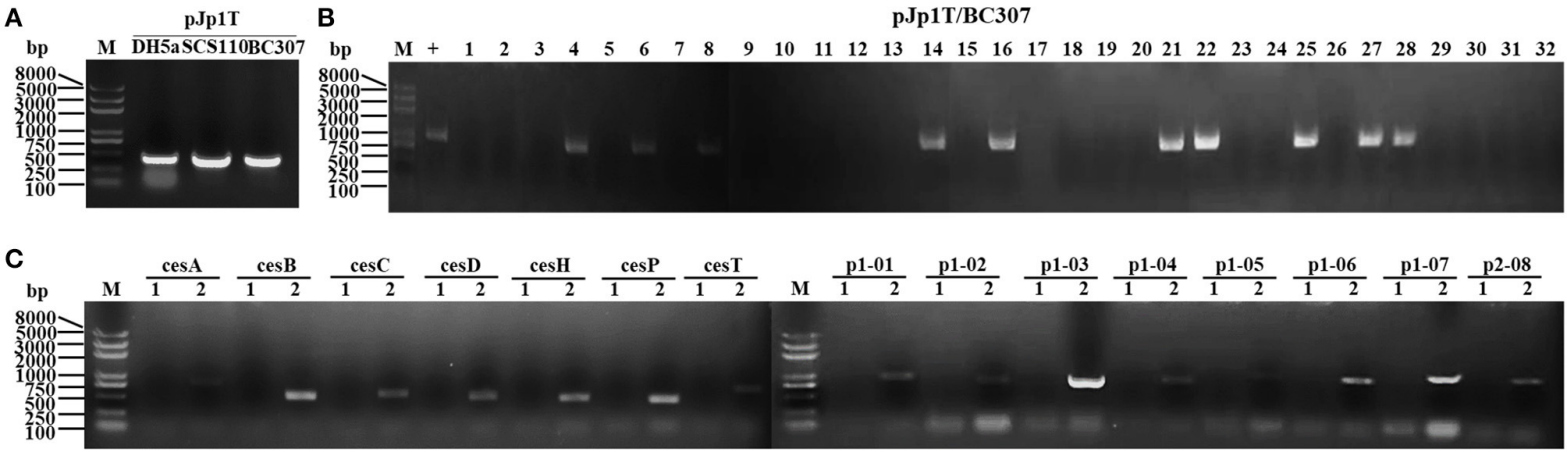
Agarose gel electrophoresis of colony PCR for the cured pCE1 plasmid from *B. cereus*. **(A)** Colony PCR to identify the strains constructed with spacer-F/R. M, *Trans*2K Plus II DNA marker. *Escherichia coli* DH5α containing the pJp1T scissors plasmid was the control. *Bacillus cereus* BC307 containing pJp1T had the same specific amplification fragment size. **(B)** Colony PCR to screen for pCE1 plasmid-cured strains with *ces*B-F/R. M, *Trans*2K Plus II DNA marker. The pCE1 plasmid-cured strains lack specific amplification fragments, and the curing efficiency was ~69% (lanes 1–3, 5, 7, 9–13, 15, 17–20, 23, 24, 26, and 29–32). **(C)** Colony PCR to confirm pCE1 plasmid curing using multiple primers. M, *Trans*2K Plus II DNA marker. *cesA, cesB*, p1-01, and p1-02 above the short line are 15 gene primers on the pCE1 plasmid. The plasmid p1-cured *B. cereus* BC307Dp1 (pCE1^−^) strain (lane 1) lacks specific amplification fragments with *ces*X primers and p1-X primers, whereas, *B. cereus* BC307, the active control (lane 2), generated specific amplification fragments.

We used colony PCR with spacer-F/R primers to identify the pJp1T scissors plasmid in *E. coli* DH5α, SCS110, and *B. cereus* BC307 transformants. The constructed strain was named pJp1T/BC307 (Figure 7A). We then used colony PCR to screen for pCE1-cured strains with *ces*B-F/R primers. Monoclonal clones lacking specific amplification fragments were possibly cured the pCE1 plasmid, with an efficiency of about 68% (22/32) (Figure 7B). We also used colony PCR with multiple primers to determine whether the pCE1 plasmid was cured from *B. cereus* BC307. The plasmid-cured pCE1 strain was designated BC307Dp1 (pCE1^−^) (Figure 7C).

### Specific Killing of *B. anthracis*

#### Growth Curve Assays

**Figure 8:**
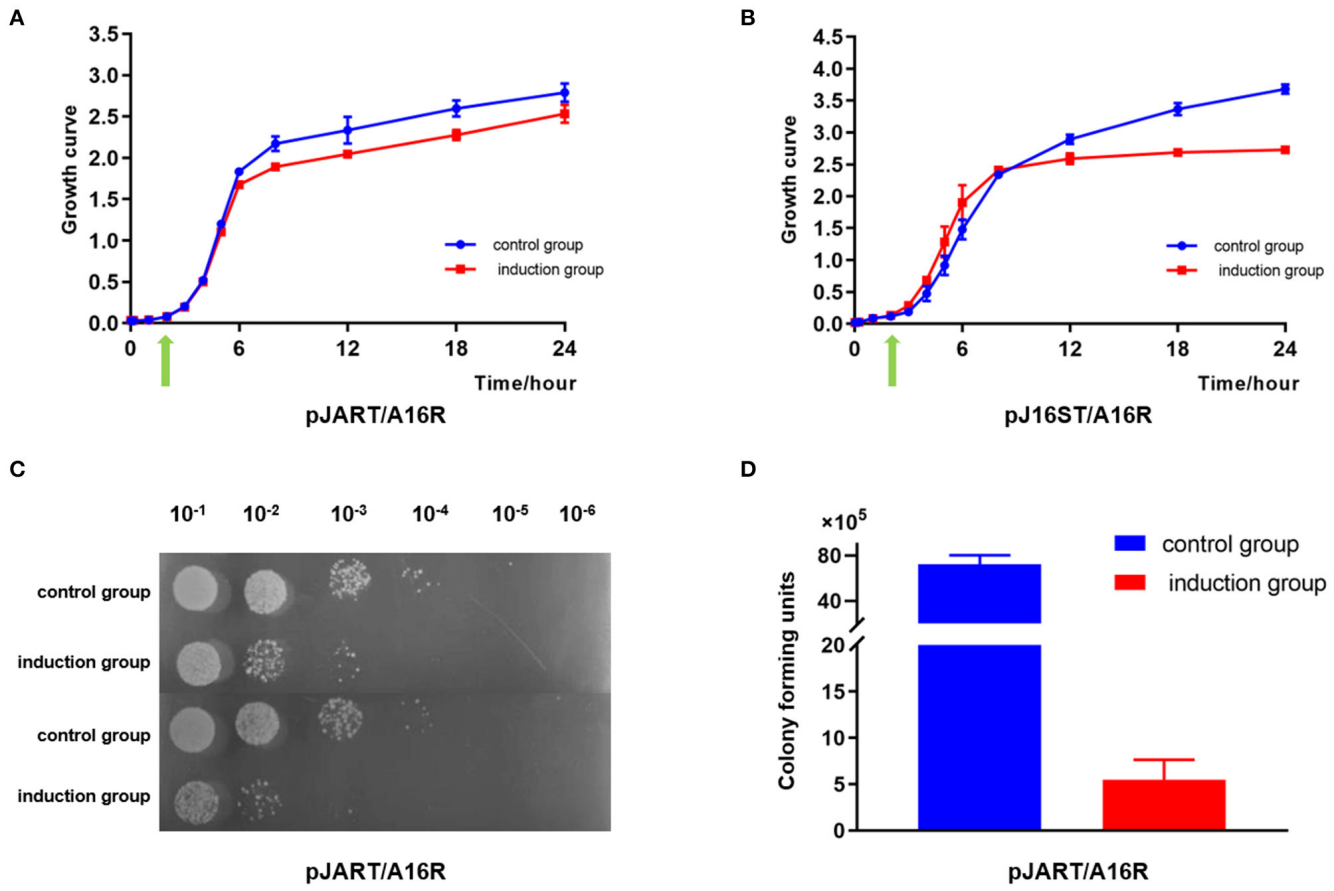
The sterilization efficiency of *B. anthracis* by using the different sgRNAs. The experimental group was cultured continuously for 3 h and 0.4% D-mannose was added (green arrow). **(A)** pJART/A16PI2 with single-site sgRNA. The growth curve from the experimental group (pJART/A16PI2 with single-site target sgRNA, with D-mannose, red line) is lower (0.3–0.4) than that of the control group (without D-mannose, blue line) throughout the stationary phase. **(B)** pJ16ST/A16PI2 with multi-site target sgRNA. The growth curve from the experimental group (pJ16ST/A16PI2 with multi-sites sgRNA, with D-mannose, red line) is lower (0.3–0.6) than that of the control group (without D-mannose, blue line) throughout the stationary phase. **(C,D)** After incubation with 0.4% D-mannose, the numbers of pJART/A16PI2 colonies were (5.5 ± 2.1) × 10^5^ CFU/mL and (72.5 ± 7.8) × 10^5^ CFU/mL in the induction group and the non-induction group. Values represent the means of at least two independent replicates. Error bars represent standard deviations.

The growth patterns of pJART/A16PI2 and pJ16ST/A16PI2 strains were continuously monitored by high-throughput real-time Microbial Analysis instrumentation. The growth of pJART/A16PI2 and pJ16ST/A16PI2 (with D-mannose) did not differ from that of the control group (without D-mannose) during the logarithmic growth phase (5–10 h), but their growth patterns decreased by OD values of 0.3–0.6 during the stationary phase (12–24 h) as compared with the control group (Figures 8A,B). After induction with 0.4% D-mannose, the numbers of pJART/A16PI2 colonies were (5.5 ± 2.1) × 10^5^ CFU/mL and (72.5 ± 7.8) × 10^5^ CFU/mL in induction group and non-induction group, respectively, with a kill efficiency of about 93% (Figures 8C,D). The results show that the sterilization efficiency between pJART/A16PI2 (with single-site target sgRNA) and pJ16ST/A16PI2 (with multi-site target sgRNA) was not obviously different, with both having some degree of sterilization efficiency. Under the condition of D-mannose induction, the breakage efficiency of the recombinant scissors plasmids pJA16sRT and pJA16sRTW, which contain two tandem sgRNAs, were the same as that of the scissor plasmids of pJ16ST and pJART, which contain only one sgRNA (see Supplementary Figure 2).

#### Specific Killing of *B. anthracis* in *B. anthracis* and *B. cereus* Mixed Cultures

**Figure 9:**
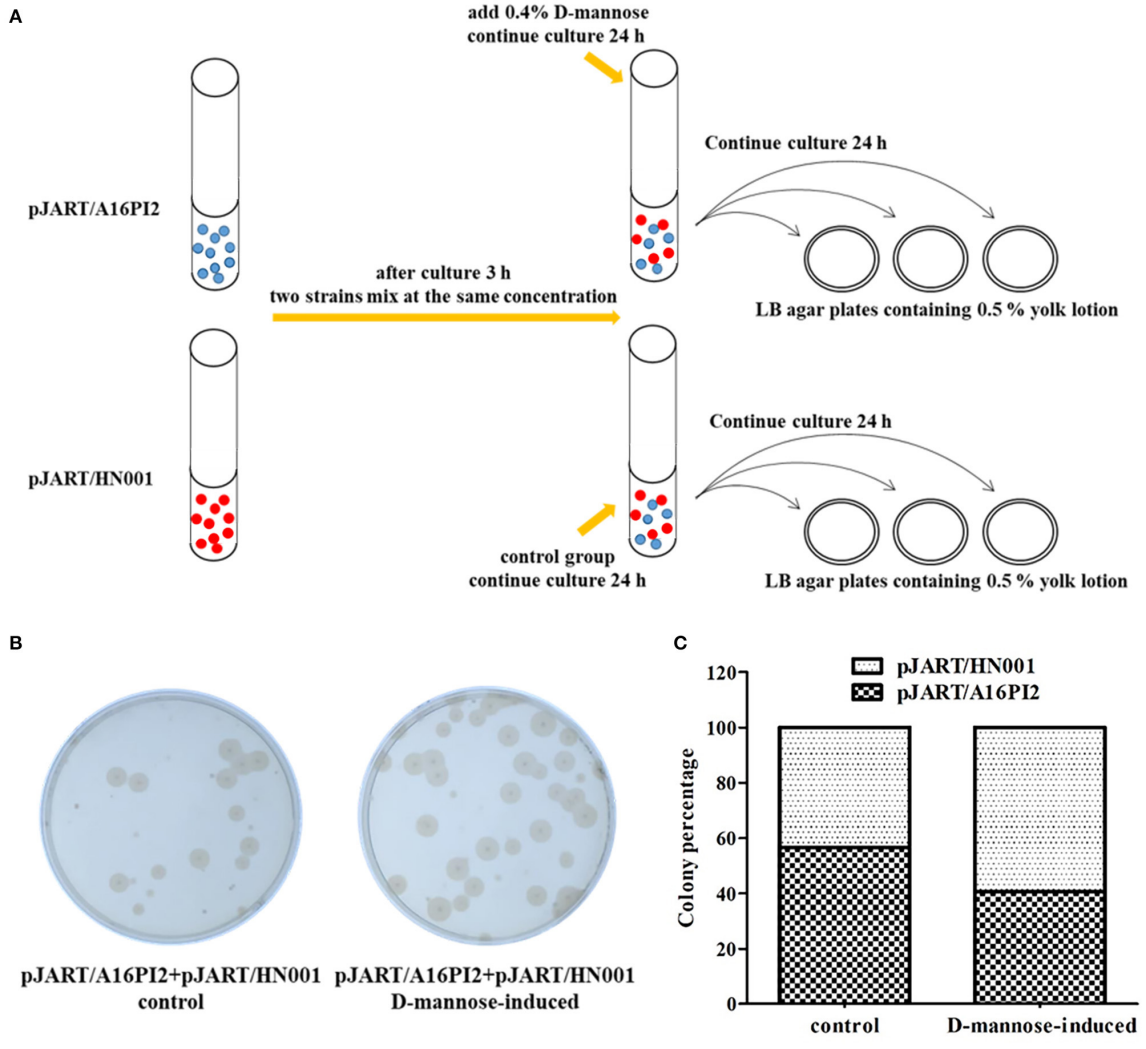
Efficiency of *B. anthracis* sterilization in a mixed culture of *B. anthracis* and *B. cereus* under D-mannose induction. **(A)** The colony counting process used for the mixed strains. Blue balls represent strain pJART/A16PI2 and red balls represent strain pJART/HN001. **(B)** pJART/A16PI2 and pJART/HN001 grew on LB agar plates (containing 0.5% yolk lotion). The percentage of *B. anthracis* A16PI2 in the D-mannose-induced group (without proteolytic rings around the colonies) was about 10-fold less than that of the control group. **(C)** Counts of *B. anthracis* in the different groups. The black-shaded block denotes *B. anthracis* pJART/A16PI2 and the white-shaded block denotes *B. cereus* pJART/HN001. *Bacillus anthracis* (black-shaded block) accounted for 40% of the bacterial percentage in the D-mannose-induced group, while *B. anthracis* accounted for 57% in the control group.

Based on the above results, the specific killing efficiency of *B. anthracis* was assessed using pJART/A16PI2 and pJART/HN001 strains with single-site sgRNA. The experiment was performed according to Figure 9A. *Bacillus cereus* was positive for lecithinase and hemolysis activity. Before D-mannose induction, the ratio of *B. anthracis* to *B. cereus* was 57%: 43%, but after mannose induction, the ratio became 40%: 60% (Figures 9B,C). These results show that the pJART plasmid transfected into *B. anthracis* has the ability to specifically kill *B. anthracis* under D-mannose-induction, and the killing efficiency of *B. anthracis* was 10–17%.

## Discussion

The principle of “plasmid incompatibility” has been used previously to guide methodology aimed at curing the large virulence plasmid in *B. anthracis*. Plasmid incompatibility is the introduction of an incompatible plasmid group into a bacterium resulting in genetic instability of the original plasmid, possibly caused by competition for the same replication or segregation sites, or from inhibition of replication initiation (Novick and Hoppensteadt, 1978; Novick et al., 2009). Thus, knowledge of the exact origin of DNA replication in the target plasmid is essential, as is the need to culture the new plasmid-containing strain for 5–10 generations to obtain a strain that repels the large virulence plasmid. The wide application of CRISPR/Cas9 gene editing technology provides researchers with a simpler, more efficient method for plasmid curing. We only need to design different N20 sequences to guide the scissors Cas9 protein to different target sites. This method is simple to use and has a good specificity. It is not necessary to know the complete sequence or its function when designing a plasmid curing protocol, and the cure efficiency is very high. In this study, the pXO1-cure efficiency was 96% when the sgRNA targeted the replication initiation region and 92% when the sgRNA targeted the non-replication initiation region. When we cured the pXO2 plasmid, the situation was much the same, with the pXO2-cure efficiency being 100% when the sgRNA targeted the replication initiation region, and 88% when the sgRNA targeted the non-replication initiation region. This indicates that there is a very slight elimination efficiency difference when the sgRNA target the replication initiation region. We also tried using a mixture of the two scissors plasmids to simultaneously eliminate both pXO1 and pXO2 virulence plasmids from *B. anthracis*, the result of which was that the single-plasmid cure rate and the double-plasmid cure rate was 29 and 14%, respectively (Table 3).

**Table 3:** Plasmid elimination rates in *B. anthracis* and *B. cereus* using the CRISPR/Cas9 system.

**Scissors Plasmids**	**Target Strain**	**Target Plasmid**	**Target ORI**	**Result**		
				**Cured**	**No-cured**	**Eliminate rate (%)**
pJO1T	A16PI2	pXO1	Yes	23	1	96
pJO1NT			No	22	24	92
pJO2T	A16Q1	PXO2	Yes	24	24	100
pJO2NT			No	21	24	88
pJO1T+pJO2T	A16	pXO1	Yes	5	12	29
		pXO2		5	12	29
		pXO1+pXO2		2	12	14
pJN1F2T	A16	pXO1	No	0	0	0
		pXO2	Yes	10	1	91
		pXO1+pXO2		0	0	0
pJF2N1T	A16	pXO1	No	11	0	100
		pXO2	Yes	0	0	0
		pXO1+pXO2		0	0	0
pJN1F2W	A16	pXO1	No	0	0	0
		pXO2	Yes	11	0	100
		pXO1+pXO2		0	0	0
pJF2N1W	A16	pXO1	No	8	3	73
		pXO2	Yes	0	0	0
		pXO1+pXO2		0	0	0
pJp1T	BC307	BC307 pCE1	No	22	10	69

We also designed a new recombinant scissors plasmid that inserted two tandem sgRNAs into the bone vector pJOE8999 to cure both pXO1 and pXO2 from wild type *B. anthracis* A16 simultaneously. According to our design, the RNA sequence N20_pXO1_gRNA-N20_pXO2_gRNA will be obtained after transcription. This RNA sequence will be treated with RNase III or other enzymes in the bacteria to obtain two independent sgRNAs: N20_pXO1_gRNA and N20_pXO2_gRNA. Under the guidance of N20_pXO1_ and N20_pXO2_, the plasmids pXO1, and pXO2 will be targeted and cleaved respectively. However, the experimental results were inconsistent with our expectations. Our experimental results showed that the recombinant scissors plasmid containing two tandem sgRNAs could not simultaneously excise two target plasmids. The second sgRNA sequence could cure the corresponding target plasmid with high efficiency, but the first sgRNA did not work in all the experiments we designed.

We presume that the enzymes required for digestion the tandem sgRNA are not worked in *B. anthracis*, resulting in the two transcribed sgRNAs always being in tandem. In this tandem sgRNA, the 3′ end of the second sgRNA can form the structure required for binding Cas9, so the plasmid targeted by the second sgRNA can be excised. However, due to the influence of the sequence of the second sgRNA at the 3′ end of the first sgRNA, the first might not be able to form the structure required for binding to Cas9, and thus cannot cut the target plasmid. This phenomenon is interesting and worthy of further investigation.

*Bacillus anthracis, B. cereus* and *B. thuringiens*is are *B. cereus* group members, and the bacteria in this group mostly contain plasmids. Many specific biochemical functions, such as toxin production and resistance to antibacterial drugs, for example, are inherited through plasmids (Helgason, 2000). When we used the CRISPR/cas9 system to eliminate the pCE1 virulence mega-plasmid from *B. cereus* BC307 by simply changing the sgRNA, we also obtained a plasmid-cured isogenic strain with a very high elimination rate (69%) (Table 3). We quickly and easily cured the plasmids from these strains through the CRISPR/Cas9 system, which provides new methods and ideas for studying virulence-related genes.

Although we were able to cure the plasmid by designing sgRNA, we did not know whether we could kill the bacteria by targeting 16sRNA or other sites on the chromosomes. Therefore, we designed two different target sequences with which to break the *B. anthracis* chromosome, and found that the sterilization efficiency of *B. anthracis* was about 93%, with no significant difference in the efficiency of multi-site and single-site targeting. At the same time, we also designed an experiment to sterilize the *B. anthracis* by two sgRNAs in tandem. The sterilization efficiency is no different from that of a single sgRNA. This result is similar to our previous results of using tandem sgRNA to simultaneously curing the *B. anthracis* plasmids pXO1 and pXO2. In the tandem sgRNA used for sterilization, only the second sgRNA might have an effect on sterilization, and the first sgRNA cannot form the required structure to bind Cas9, so it has no effect on killing bacteria.

Thus, our results have shown that the CRISPR-Cas9 system can be useful for gene editing in *B. cereus* group strains, and that it can cure plasmids simply and efficiently. The cure efficiency might differ depending on the N20 target sequences that are chosen. Our results indicate that the plasmid elimination rate is only slightly higher when the replication initiation region is the sgRNA target, compared with the non-replication initiation region. CRISPR targeting of virulence genes can select for the loss of CRISPR function during infection, when the acquisition of those genes is under strong selective pressure (Jiang et al., 2013; Gomaa et al., 2014). Mutations in the replication initiation region have a more negative effect than mutations in the non-replication replication region, so we speculate that the Cas9 system has higher levels of off-target mutagenesis in the non-replication initiation region than in the replication initiation region, making the cure efficiency of the non-replication initiation region lower. With a lower efficiency of chromosome breakage, we speculate that off-target mutagenesis of chromosome and the self-repair mechanism in *B. anthracis* plays a key role in bacterial survival when faced with an external killing pressure. On the other side, it is also likely that the curing site we chose may not be suitable. The high efficiency of plasmid curing may only cause a change in pathogenicity, antibiotic resistance, or other metabolic processes when the selection pressure is relatively small. Furthermore, we only induced one generation of bacteria in our experiment, and increasing the induction period may increase the killing rate.

## Data Availability Statement

All datasets generated for this study are included in the article/Supplementary Material.

## Author Contributions

DW, XL, and HW designed the research study. XW, YL, SiW, QZ, EF, LZ, and CP performed all the experiments. XW, DW, and XL analyzed the data. XW, XL, ShW, and DW wrote the manuscript. All authors have reviewed the final manuscript.

## Conflict of Interest

The authors declare that the research was conducted in the absence of any commercial or financial relationships that could be construed as a potential conflict of interest.
