# Supplementary material for: Application of CRISPR/Cas9 System for Plasmid Elimination and Bacterial Killing of Bacillus cereus Group Strains
Source: Front Microbiol. 2021 Jun 10;12:536357. doi: 10.3389/fmicb.2021.536357 (PMC8222586; doi:10.3389/fmicb.2021.536357)
Supplement: Supplementary file 1 [file Data_Sheet_1.docx]

**Supporting Information**

**S1 The constructing process of the recombinant plasmids**

The primers were diluted in water to 50μmol, and 9μL of each primer and 2μL of T4 ligase buffer was used. The primers were heated to 95^o^C for 1min, and then naturally cooled to room temperature (Table 2). pJOE8999 ([Altenbuchner, 2016](#_ENREF_3)) was digested with *Bsa*I restriction endonuclease at 37^o^C for 30–60min, and the digested linearized products were recovered. The double-stranded N20 oligonucleotides were ligated to their corresponding linearized plasmid vectors using 7μL of the double-stranded N20 oligonucleotide, 1μL of the linearized pJOE8999 plasmid, 1μL of T4 ligase buffer, 1μL of T4 ligase, with ligation at 22^o^C for 30min. The ligation products were transformed into *E. coli* DH5α, white colonies were selected on LB agar (, 20μg/ml X-gal), and the spacer-F/R (Table 2) was used for amplifying and sequencing the plasmids to confirm that the N20 sequences had inserted into pJOE8999 at the 5'end of the sgRNA.

**S2 Specific Killing efficiency of** ***B. anthracis* using the plasmid with two sgRNAs of 16ST and ART inserting into pJOE8999 in tandem**

After incubation with 0.4% D-mannose, the numbers of pJ16SRT/A16PI2 colonies were (13.7 ± 4.5)×10^5^ CFU/mL and (96.7 ± 11.0) ×10^5^ CFU/mL in induction group and non-induction group, respectively, with a kill efficiency of about 86%. And the number of pJ16SRTW/A16PI2 colonies under the incubation was (15.0 ± 3.0) ×10^5^ CFU/mL, and was less than (100.3 ± 3.2) ×10^5^ CFU/mL in the control group, with a kill efficiency of about 85%. These results show that the sterilized efficiency of plasmid containing two tandem sgRNAs targeting different sites is above 85%. The sterilized efficiency A16PI harboring the 16ST and ART tandemly ligated on the pJOE8999 plasmid is similar to that of the pJ16ST/A16PI2, pJART/A16PI2 and pJAoriT/A16PI2 under D-mannose-induction.


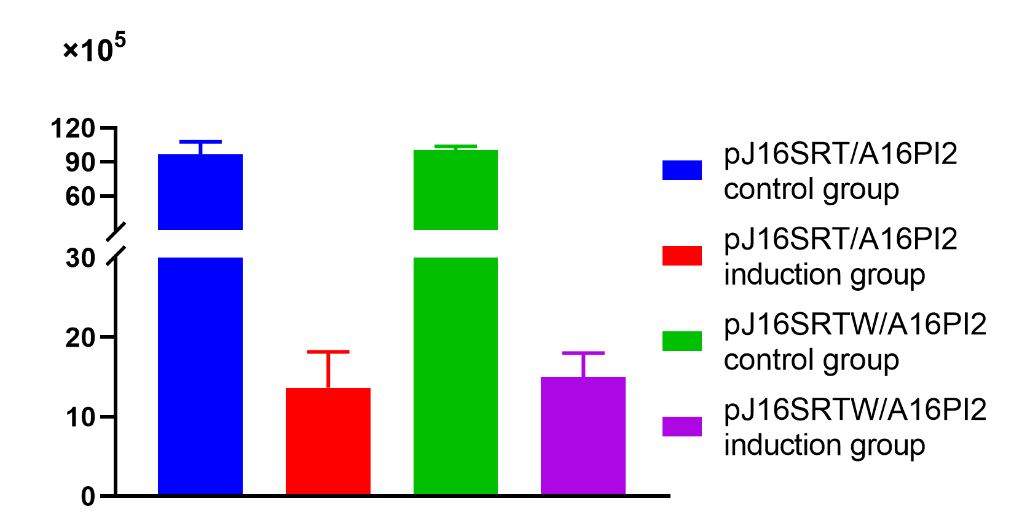


Fig. S2 Efficiency of *B. anthracis* sterilization using the plasmid ligated by 16ST and ART together. Values represent the means of at least three independent replicates. Error bars represent standard deviations.
